# MRI features of joint capsule-originating hamartomas in a pediatric patient with tuberous sclerosis: a novel case report

**DOI:** 10.1007/s00256-025-05122-x

**Published:** 2026-01-14

**Authors:** Yusuf Yahsi, Rodi Ertogrul, Hayri Ogul

**Affiliations:** 1 Department of Orthopedic Surgery, Medical Faculty, Istanbul Medipol University, Istanbul, Turkey; 2 Department of Radiology, Medical Faculty, Istanbul Medipol University, Istanbul, Turkey

**Keywords:** Tuberous sclerosis, Articular, Hamartoma, MR imaging

## Abstract

Hamartomas are well-recognized benign growths associated with tuberous sclerosis complex (TSC), occurring frequently in the skin, central nervous system, and kidneys. Although musculoskeletal manifestations have been documented, intra-articular hamartomas originating specifically from the joint capsule have not been previously reported to the best of our knowledge. We present a unique case involving a 9-year-old female with genetically confirmed TSC who exhibited multiple intra-articular hamartomatous lesions originating from the joint capsules of the hip and knee. These lesions were distinctly visible on magnetic resonance imaging (MRI), displaying signal characteristics consistent with hamartomas, and they were confirmed by histopathological analysis. This underscores the importance of considering capsular hamartomas in the differential diagnosis of intra-articular masses in TSC and highlights the diagnostic value of MRI in identifying such rare manifestations.

## Introduction

Tuberous sclerosis complex (TSC) is an uncommon autosomal dominant neurocutaneous disorder resulting from pathogenic mutations in either the TSC1 or TSC2 genes, leading to dysregulation and hyperactivation of the mammalian target of rapamycin (mTOR) signaling pathway [1]. This facilitates the formation of hamartomatous lesions in various organ systems, including the brain, kidneys, heart, skin, and eyes. Although the classical clinical triad of seizures, intellectual disability, and facial angiofibromas is well established, the phenotypic expression of TSC is considerably variable, exhibiting a wide array of clinical manifestations [2]. In recent years, the prevalence of musculoskeletal involvement in TSC has been a subject of considerable interest, particularly in view of the reports of sclerotic bone lesions and soft tissue tumors [3].

Although soft tissue hamartomas associated with TSC are clearly recorded [4], primarily in the skin and subcutaneous layers, the presence of intra-articular hamartomas, particularly originating from joint capsule structures, has not been previously reported. Our patient represents a rare case involving multiple intra-articular hamartomatous lesions originating from the joint capsules of the hip and knee. Magnetic resonance imaging (MRI) revealed well-defined intra-articular lesions with signal characteristics suggestive of hamartomatous tissue, and genetic analysis confirmed a pathogenic heterozygous c.3282C>T (p.Gln1094) mutation in the TSC2 gene. Concurrent radiography and targeted musculoskeletal ultrasonography of the affected joints demonstrated no additional diagnostic findings beyond those identified on MRI. This case broadens the current understanding of musculoskeletal involvement in TSC, highlighting the importance of considering lesions related to the joint capsule in the differential diagnosis of intra-articular masses in affected individuals.

## Case report

A 9-year-old female with both genetically and clinically confirmed TSC was referred to the orthopedic outpatient department for assessment of asymptomatic masses around the left knee and left hip. A diagnosis of TSC was established at 3 months of age, following the emergence of hypomelanotic macules and the onset of epileptic seizures. Cranial MRI identified cortical tubers and subependymal nodules, and subsequent genetic analysis confirmed a heterozygous pathogenic variant c.3282C>T (p.Gln1094*) in the TSC2 gene, resulting in a premature stop codon. Over time, the patient developed multiple systemic manifestations, including renal angiomyolipomas, subcutaneous fibromas, and cutaneous angiofibromas, aligning with the TSC clinical spectrum.

At the age of 3 years, an MRI of the left knee revealed, well-defined, lobulated soft tissue mass (measuring 90 × 70 × 75 mm) originating from the anterior joint capsule, extending posteriorly into the joint space. The mass exhibited predominantly fat signal intensity and internal tubular structures, and it was identified as a hamartoma. Partial resection of the mass was performed, and histopathological analysis of the excised specimen revealed a composition characteristic of a mature hamartoma, comprising adipose, fibrous, and vascular elements and having no indications of atypia or malignancy. A histology sample was not available because the surgery was performed at another hospital.

**Fig. 1:**
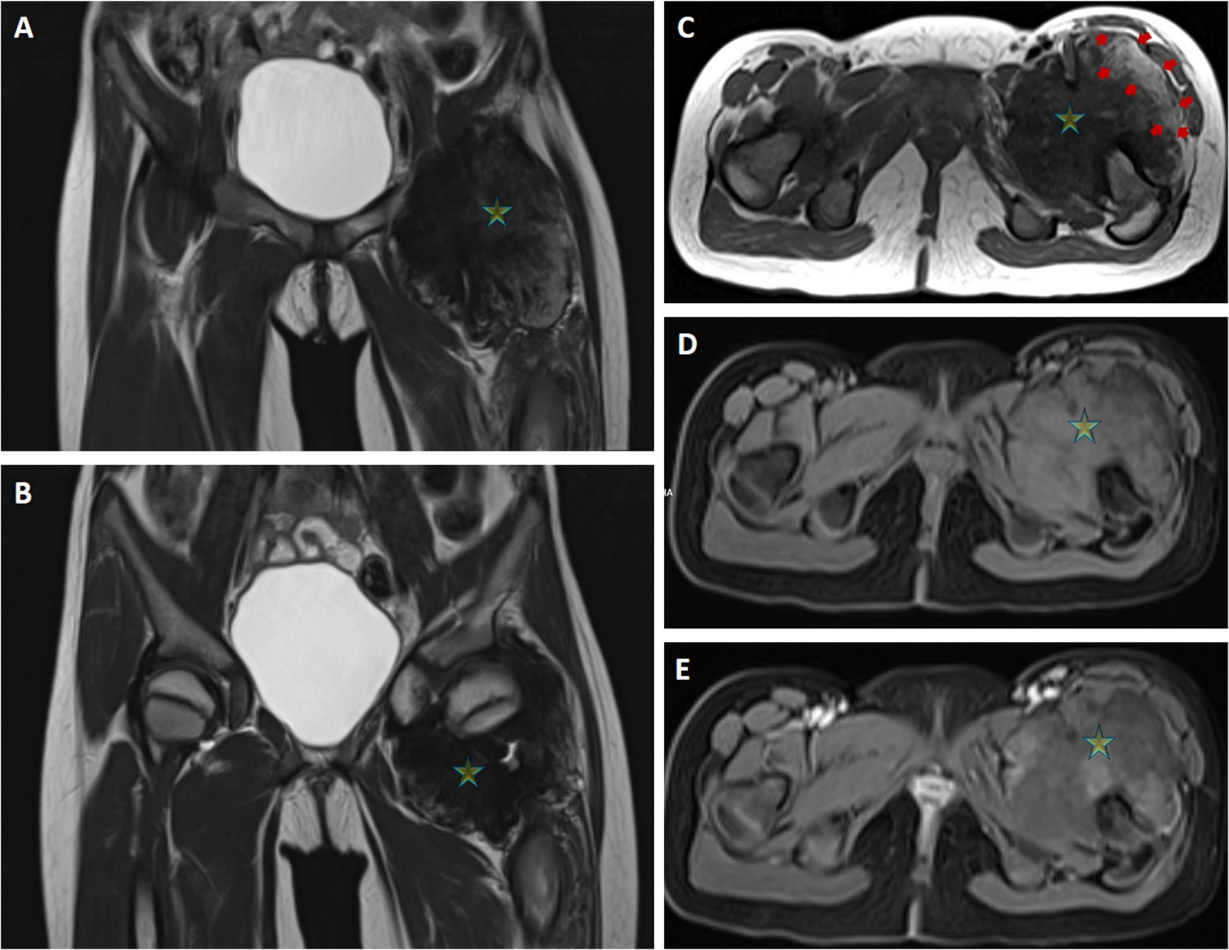
Coronal non-fat-suppressed T2-weighted (**A** and **B**) and axial (**C**) non-fat-suppressed T1-weighted magnetic resonance (MR) images of the left hip show a predominantly hypointense intra-articular mass (star) containing high-signal foci (red arrows) representing a fibrolipomatous component extending into the anterior muscular plane. Pre- and post-contrast fat-suppressed T1-weighted MR images (**D** and **E**) show minimal contrast enhancement and reveal signal loss in the fat-containing components of the mass

**Fig. 2:**
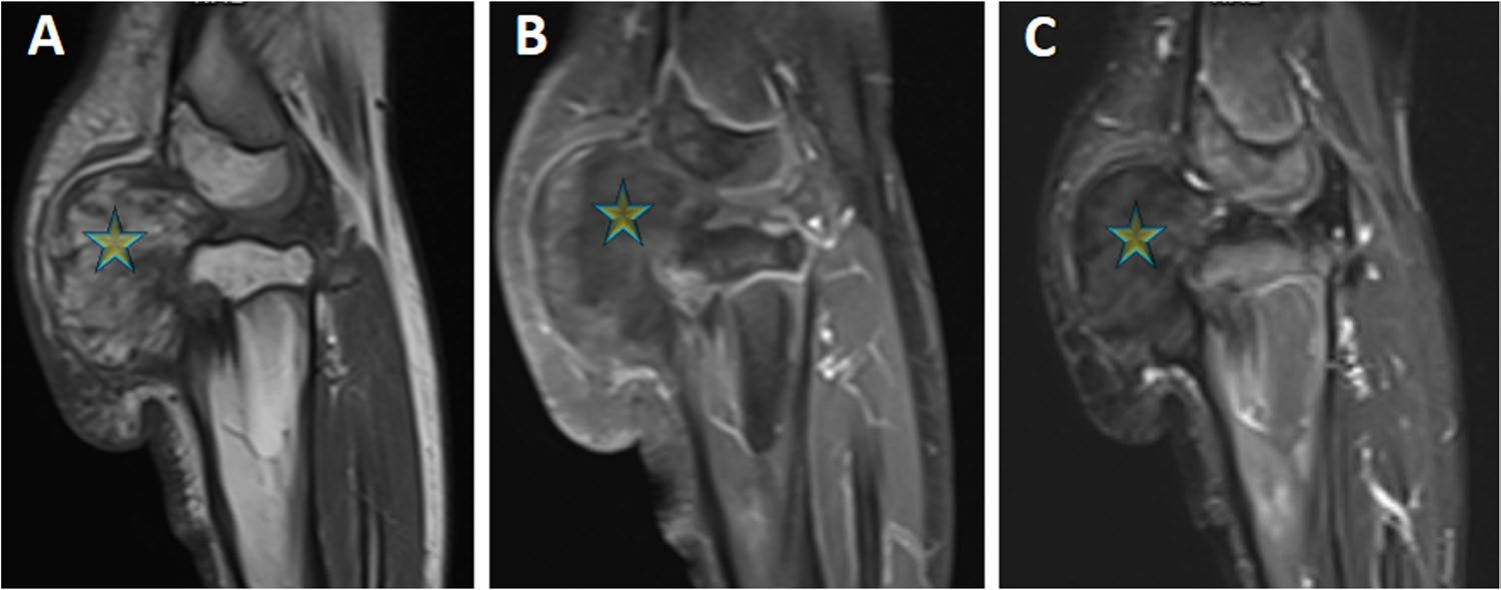
A sagittal non-fat-suppressed T1-weighted MR image (**A**) reveals a residual or recurrent intra-articular heterogenous-hyperintense lesion (star). Post-contrast fat-suppressed sagittal T1-weighted image (**B**) shows a minimal contrast enhancement (particularly anteriorly) and signal loss in the fat-containing components. Sagittal fat-suppressed T2-weighted MR image (**C**) also demonstrates signal loss in the fat-containing components

At 9 years old, the patient returned with swelling in the left knee and left hip, and an MRI of the latter showed a lobulated, capsular-based soft tissue mass measuring 74 × 80 × 95 mm, characterized by a fibrolipomatous composition extending into the anterior muscular plane (Fig. 1). The lesion was predominantly hypointense on T1- and T2-weighted MRI images, but there were high-signal foci within the lesion on non-fat-suppressed T1- and T2-weighted images. MRI features of the lesions in a patient with known TSC suggested a hamartomatous lesion; simultaneously, the MRI of the left knee revealed a residual or recurrent lesion with similar imaging characteristics (Fig. 2). Mild synovial enhancement and effusion in the tibiotalar joint were also observed.

## Discussion

TSC represents a genetically mediated multisystem disorder, arising from loss-of-function mutations in either the TSC1 or TSC2 gene. This genetic alteration leads to the continuous activation and dysregulation of the mTOR signaling pathway, promoting hamartomatous growth across a wide array of tissues, most notably within the brain, kidneys, skin, heart, and lungs. Despite osseous and soft tissue manifestations, such as sclerotic bone lesions and subcutaneous tumors, having been documented in TSC, the intra-articular space has not yet been reported as a site of hamartomatous involvement [5–7].

This case describes the presence of hamartomatous lesions originating specifically from joint capsules in a pediatric TSC patient not previously described to the best of our knowledge. The anatomical location is particularly noteworthy due to the mesenchymal origin of the joint capsule, a tissue compartment known for its sensitivity to mTOR pathway activity [8]. While capsular-based soft tissue masses are relatively common in various rheumatologic and neoplastic conditions, their identification as hamartomas in the TSC context has broadened the recognized distribution of disease-related tissue overgrowth [9].

From an imaging perspective, the lesions in question demonstrated MR findings compatible with a benign soft tissue process rather than features specific for a hamartoma or diagnostic of TSC. These more benign MR characteristics included the presence of a lobulated structure, relative hypointensity on T2-weighted images, internal septations, and the absence of aggressive characteristics, such as diffusion restriction, cortical bone destruction, or soft tissue invasion. When interpreted in conjunction with the established clinical diagnosis of TSC, these features supported the consideration of a hamartomatous lesion. These observations play a critical role in the clinical decision-making process, enabling radiologists and orthopedic surgeons to consider conservative or observational management strategies, particularly for patients who are asymptomatic. Given the systemic and potentially multifocal nature of TSC, whole-body MRI may provide comprehensive surveillance analogous to that recommended for cancer-predisposing syndromes. 

Histopathological validation of a mature hamartoma is characterized by fibrous, adipose, and vascular elements, confirming the non-neoplastic nature of these lesions, which supports with the imaging characteristics noted during follow-up. Although benign, these masses have the potential to imitate various other intra-articular pathologies. Differential diagnoses for capsular-based joint lesions include tenosynovial giant cell tumors, synovial chondromatosis, lipoma arborescens, and intra-articular lipomas [10]. Thus, an understanding of the TSC-specific pathology in such cases can inform appropriate diagnostic approaches and prevent unwarranted biopsy or surgical removal.

Research on genotype–phenotype associations has identified TSC2 mutations are linked to more severe and early-onset systemic manifestations compared to TSC1 mutations [11]. The specific nonsense mutation confirmed in the discussed patient (c.3282C>T, p.Gln1094*) resulted in a premature stop codon and a truncated protein, confirming the high disease burden, as well as multi-organ involvement from infancy [12]. The occurrence of bilateral joint capsular hamartomas may therefore represent an extreme end of the phenotypic spectrum, consistent with previous studies demonstrating an increased lesion burden in patients with TSC2 mutations [13].

The pathophysiological mechanism underlying the formation of capsular hamartomas in TSC might involve localized fibroblastic proliferation mediated by mTOR within synovial and capsular tissues, both of which originate embryologically from mesenchymal tissue [14]. Further, given the structural continuity between the capsule and surrounding connective tissue planes, proliferative activity in this region may be propelled by the same dysregulated cell signaling pathways responsible for cortical tubers in the central nervous system or angiomyolipomas in the kidneys [15]. The bilateral distribution and recurrence pattern further suggest a systemic origin rather than a localized reactive process [16].

Capsular hamartomas may be under-recognized within the TSC population, particularly in asymptomatic individuals or in those who have not undergone targeted joint imaging. As cross-sectional imaging becomes more frequently used in pediatric patients with complex genetic syndromes, similar lesions may be incidentally identified more often, emphasizing the importance of awareness to prevent misclassification as neoplastic processes.

Furthermore, longitudinal MRI surveillance may hold significant value in selected patients with a high TSC burden, particularly those with early-onset disease or TSC2 mutations. The integration of musculoskeletal—and specifically articular—assessment into TSC follow-up protocols has the potential to enhance diagnostic confidence and reduce unnecessary interventions, thereby reinforcing the role of MRI in the comprehensive care of these patients [17].

In summary, this case contributes a new perspective of the musculoskeletal characteristics observed in tuberous sclerosis. To our knowledge, this represents the first documented instance of hamartomas originating from a joint capsule in an individual with TSC. The findings underscore the need to integrate genetic, radiological, and clinical data into the diagnosis and management of rare disease manifestations. Additional documentation of comparable cases may help clarify the prevalence and clinical relevance of articular hamartomas in TSC, contributing to the refinement of guidelines for surveillance and intervention.

## Data Availability

The data that support the findings of this study are not openly available due to reasons of sensitivity of human data and are available from the corresponding author upon reasonable request.
